# Distance sampling surveys reveal 17 million vertebrates directly killed by the 2020’s wildfires in the Pantanal, Brazil

**DOI:** 10.1038/s41598-021-02844-5

**Published:** 2021-12-16

**Authors:** Walfrido Moraes Tomas, Christian Niel Berlinck, Rafael Morais Chiaravalloti, Gabriel Paganini Faggioni, Christine Strüssmann, Renata Libonati, Carlos Roberto Abrahão, Gabriela do Valle Alvarenga, Ana Elisa de Faria Bacellar, Flávia Regina de Queiroz Batista, Thainan Silva Bornato, André Restel Camilo, Judite Castedo, Adriana Maria Espinóza Fernando, Gabriel Oliveira de Freitas, Carolina Martins Garcia, Henrique Santos Gonçalves, Mariella Butti de Freitas Guilherme, Viviane Maria Guedes Layme, Ana Paula Gomes Lustosa, Ailton Carneiro De Oliveira, Maxwell da Rosa Oliveira, Alexandre de Matos Martins Pereira, Julia Abrantes Rodrigues, Thiago Borges Fernandes Semedo, Rafael Augusto Ducel de Souza, Fernando Rodrigo Tortato, Diego Francis Passos Viana, Luciana Vicente-Silva, Ronaldo Morato

**Affiliations:** 1 Embrapa Pantanal, Laboratório de Vida Selvagem, Corumbá, MS 79320-900 Brazil; 2 Instituto Chico Mendes de Conservação E Biodiversidade - ICMbio, Centro Nacional de Pesquisa e Conservação de Mamíferos Carnívoros, 12.952-011, Atibaia, SP Brazil; 3 Smithsonian Conservation Biology Institute, Front Royal, VA 22630 USA; 4 Instituto Federal de Mato Grosso do Sul - IFMS, Campus Corumbá, Corumbá, MS 79310-110 Brazil; 5 Faculdade de Medicina Veterinária, Universidade Federal de Mato Grosso - UFMT, Cuiabá, MT 78060-900 Brazil; 6 Instituto de Geociências, Universidade Federal do Rio de Janeiro - UFRJ, Rio de Janeiro, RJ 21941-916 Brazil; 7 Instituto Chico Mendes de Conservação E Biodiversidade - ICMBio, Centro Nacional de Pesquisa e Conservação de Répteis E Anfíbios, Goiânia, GO 74605-090 Brazil; 8 Instituto de Biociências, Universidade Federal de Mato Grosso - UFMT, Cuiabá, MT 78060-900 Brazil; 9 Centro Nacional de Avaliação da Biodiversidade e de Pesquisa e Conservação Do Cerrado, Instituto Chico Mendes de Conservação e Biodiversidade - ICMBio, Brasília, DF 70635-800 Brazil; 10 Instituto Brasileiro de Meio Ambiente e Recursos Naturais Renováveis - IBAMA, Corumbá, MS 79331-150 Brazil; 11 Universidade Federal de Mato Grosso Do Sul - UFMS, Campus Pantanal, Corumbá, MS 79304-902 Brazil; 12 Universidade Federal de Mato Grosso Do Sul - UFMS, campus Campo Grande, Campo Grande, MS 79070-900 Brazil; 13 Fundação de Meio Ambiente do Pantanal, Corumbá, MS 79331-100 Brazil; 14 Sauá Consultoria Ambiental, Corumbá, MS 79332-210 Brazil; 15 Laboratório de Ecologia de Mamíferos, Universidade Federal de Mato Grosso - UFMT, Cuiabá, MT 78060-900 Brazil; 16 Centro de Pesquisa e Conservação de Aves Silvestres, Instituto Chico Mendes de Conservação e Biodiversidade - ICMBio, Brasília, DF 70635.800 Brazil; 17 Instituto Brasileiro de Meio Ambiente e Recursos Naturais Renováveis - IBAMA, Centro Nacional de Prevenção e Combate Aos Incêndios Florestais, Campo Grande, MS 79020-230 Brazil; 18 Instituto Nacional de Pesquisa do Pantanal - INPP, Museu Paraense Emílio Goeldi, Cuiabá, MT 78060-900 Brazil; 19 Bioma Meio Ambiente, Nova Lima, MG 34.006-042 Brazil; 20 Panthera, 8 West 40th Street, 18th Floor, New York, NY 10018 USA; 21 Instituto Homem Pantaneiro - IHP, Corumbá, MS 79331-150 Brazil

**Keywords:** Biodiversity, Fire ecology, Wetlands ecology

## Abstract

Anthropogenic factors have significantly influenced the frequency, duration, and intensity of meteorological drought in many regions of the globe, and the increased frequency of wildfires is among the most visible consequences of human-induced climate change. Despite the fire role in determining biodiversity outcomes in different ecosystems, wildfires can cause negative impacts on wildlife. We conducted ground surveys along line transects to estimate the first-order impact of the 2020 wildfires on vertebrates in the Pantanal wetland, Brazil. We adopted the distance sampling technique to estimate the densities and the number of dead vertebrates in the 39,030 square kilometers affected by fire. Our estimates indicate that at least 16.952 million vertebrates were killed immediately by the fires in the Pantanal, demonstrating the impact of such an event in wet savanna ecosystems. The Pantanal case also reminds us that the cumulative impact of widespread burning would be catastrophic, as fire recurrence may lead to the impoverishment of ecosystems and the disruption of their functioning. To overcome this unsustainable scenario, it is necessary to establish proper biomass fuel management to avoid cumulative impacts caused by fire over biodiversity and ecosystem services.

## Introduction

Anthropogenic factors have significantly influenced the increasing frequency, duration, and intensity of meteorological drought in many regions of the globe^1,2^, challenging the sustainability of life on Earth. Extreme droughts caused by human-induced climate change have been pointed out as the global-scale determinant of the observed increasing wildfire occurrence^3,4^. In fact, during the last few years we have been witnessing an astonishing increase in intensity and frequency of wildfires, leading to a globally unprecedented amount of burnt area^3,5,6^. However, the impacts of wildfire on wildlife are still poorly known, preventing our better understanding of the cumulative effects on the ecosystem functioning.

Besides the climate change implications for the wildfire increased frequency, many ongoing drivers at regional scales are to blame as well, including short to long-term anthropogenic activities such as deforestation, incorrect ignition and use of fire, absence of or inadequate landscape management strategies, vegetation encroachment, increased need of fire as management tool, and release of greenhouse gases which, in turn, contributes to climate change^7–12^. In fact, during the last few years we have been witnessing an astonishing increase in intensity and frequency of wildfires, leading to a globally unprecedented amount of burnt area^3,5,6^.

Seasonal wet-dry ecosystems, such as wet savannas, are particularly vulnerable to burning due to the higher vegetational load sustained by flood-induced fertility that makes these areas susceptible to burning during dry season^6,13,14^. Recently, among the most dramatic wildfire events, stands out the recorded widespread burning that hit the 170,000 km^2^ Pantanal wetland, in the center of South America, which may be characterized as an extreme wildfire event (EWE)^15^. While fires burned 16,210 km^2^ of the Brazilian portion of the Pantanal in 2019, astonishing 39,030 km^2^ burned in 2020^16^.The large quantities of organic matter accumulated in lower, long-lasting flood areas covered by dense aquatic plant communities and floating mats, as well as in the open grasslands subjected to vegetation encroachment, created the scenario for the catastrophic 2020 wildfire in the Pantanal^17,18^.

The scenarios of climate change for the Pantanal region indicate decrease in the amount of rainfall, higher temperatures, and higher frequency of extreme climate events^19^. Fire is an evolutionary driver that shapes landscapes, biodiversity, human behavior, and the dynamics of socio-ecological systems worldwide^20,21^, and a natural and important component in savanna ecosystems worldwide^22,23^. Among these savannas, the Brazilian Cerrado and Pantanal wetland have been influenced by fire since before human arrival^24,25^, and are considered fire-dependent ecosystems^20^. However, we still need a better understanding of the impact caused by wildfires on the ecological functions and ecosystem services provided by wildlife. Additionally, this understanding is relevant for the process of sensitization of landowners, decision-makers, and the whole society towards the necessity of an integrated fire management strategy for the region.

The effects of fire on wildlife populations are generally classified as first-order (direct or immediate), second-order (indirect), and evolutionary effects from fire history^26,27^. Experiences on estimating impacts of large-scale wildfire on wildlife are still scarce, and often do not separate mortality from other outcomes from this type of disturbance. Usually, estimates have been assessed based on known population densities or estimated biomass, depending on the available knowledge on specific species or species groups^28–30^. Although these assessments may be considered valid attempts of estimating the number of animals killed by fire, they are not based on direct carcass counts.

This paper focuses on estimating the deaths among vertebrates directly caused by wildfires, based on data collected in the field by accounting for carcasses up to 48 h after fire events in the Pantanal wetland, Brazil. With this report we want to contribute to the increase of awareness in society regarding the impact of such events on wildlife, as the scenario of climate change poses to humanity perhaps its major challenge in history. The estimates we present, besides its direct accounting in the field, may help to evaluate the potential cumulative impact of eventually repeated wildfires in ecosystems, as this is a plausible scenario posed by climate change.

## Results

**Figure 1:**
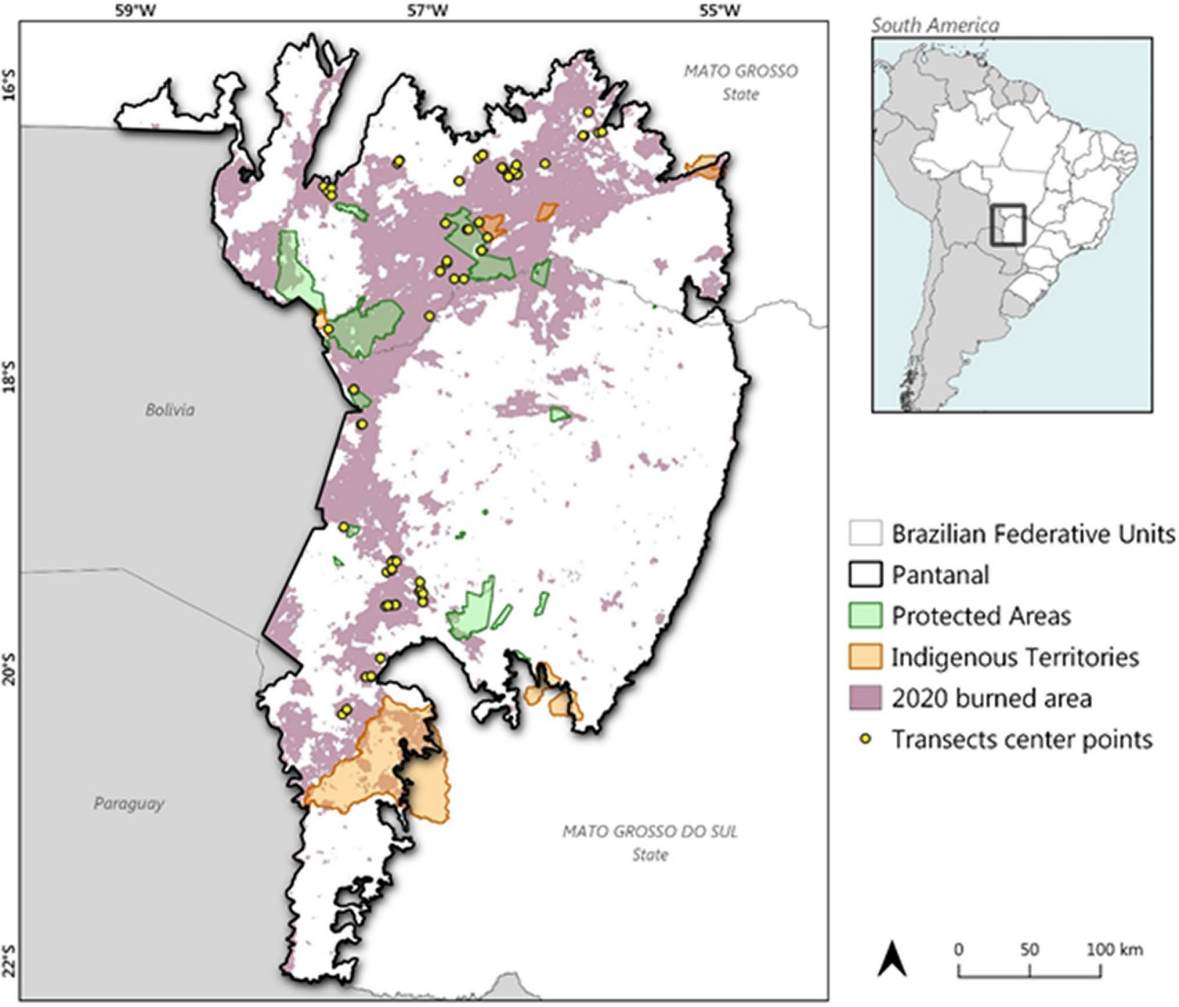
Distribution of surveyed locations (transects) to estimate first order vertebrate mortality in the area hit by wildfires in the Brazilian portion of Pantanal wetland in 2020. Burned area source: Laboratory for Environmental Satellite Applications (LASA), 2020 available at https://alarmes.lasa.ufrj.br/login.

Our survey in the Pantanal is the first found in the literature that applies distance sampling to account for animals killed by wildfires. We covered 126 line transects distributed from northern to southern Pantanal wetland (Fig. 1), totalizing 114.43 km, from which 302 records of dead vertebrates were obtained. The effectively sampled strip along transects were estimated to be 2.72 ± 0.21 m wide for small vertebrates (CV = 7.69) and 7.28 ± 1.33 m wide for medium to large vertebrates (CV = 18.26), and the overall detection probability was 0.108 ± 0.084 and 0.29 ± 0.053, respectively. The fitted probability model of the distribution of both small vertebrates and medium to large vertebrates was a Negative Exponential key, k(y) = Exp(−y/A(1)) with simple polynomial adjustments of order 2, 4, while for large vertebrates it was k(y) = Exp(−y/A(2)). As a result, we estimated that 16,009,000 ± 2,802,300 small vertebrates were killed by the wildfires, as well as 943,830 ± 252,740 medium to large vertebrates, in the 39,030 km^2^ burnt area from January to November 2020 (Table 1). In total, a pooled estimate of 16,952,000 (217.17 vertebrates per km^2^) vertebrates died due to direct effect of the 2020 wildfires in the wetland (Table 1).

**Table 1:** Estimates of density (D: carcasses per km^2^) and number of dead vertebrates (N), their standard errors, coefficient of variation (CV), and confidence interval (CI), by body size group and pooled data, where small-sized vertebrates (S): < 2 kg; medium-large sized vertebrates (L): 2 kg and over. Estimates were, obtained by analyzing distance sampling data in the area hit by wildfires in the Brazilian portion of the Pantanal wetland in 2020.

	Parameter	Point estimate	CV	95% CI (in millions)
S	D	410.16 ± 71.80	17.50	11.36—22.55
	N	16,009,000 ± 2,802,300	17.50	
L	D	24.18 ± 6.48	26,78	0.56—1.59
	N	943,830 ± 252,740	26,78	
Pooled	D	217.17	16.60	12.46—23.47
	N	16,952,000	16.60	

When considering the pooled estimate of deaths caused by the wildfires, the dead animals most frequently detected in our surveys pertained to the subgroups of small snakes, small birds, medium-large birds, and small rodents (Table 2). However, when within group composition was examined, the number of large lizards, artiodactyls, and primates were also noteworthy (Table 2). Despite the poor condition of most carcasses, most specimens were identified to the species or genus level. Those included one tortoise, five amphibian, three small lizard, two large lizard, one anaconda, nine small snake, one caiman, 11 small bird, seven medium to large bird, two small marsupial, two armadillo, one anteater, three primate, two small rodent, three medium to large rodent, and two ungulate species. Additionally, we identified the carcasses as pertaining to 55 different taxonomic entities at least to the Family level, from which 53 were identified at least to the Genus level (table S1, in the Supplementary Information).

**Table 2:** Descending order of the estimated number of dead animals in sub-groups within the small (Small) and the medium to large (Large) vertebrate groups during the wildfires that hit the Brazilian portion of Pantanal wetland in 2020.

Group	Sub-groups	% of Pooled records	Estimated number*	% of within group records	Within group estimated number**
Small	Small snakes^s^	55.4	9,391,408	65.2	9,957,598
	Small rodents^s^	19.4	3,288,688	22.8	3,650,052
	Small birds^s^	5.8	983,216	6.8	1,088,612
	Small lizards^s^	1.7	288,184	2.0	320,180
	Amphibians^s^	1.4	237,328	1.6	256,144
	Marsupials^s^	1.0	169,520	1.2	192,108
Large	Medium-large birds	3.4	576,368	22.7	214,249
	Ungulates	2.7	457,704	18.2	171,777
	Primates	2.7	457,704	18.2	171,777
	Medium-large rodents	2.4	406,848	15.9	150,069
	Caiman	1.4	237,328	9.1	85,888
	Anteaters	1.3	220,376	6.1	57,574
	Large lizards	0.7	118,664	4.5	42,472
	Chelonians	0.3	50,856	0.4	3,775
	Armadillos^s^	0.3	50,856	2.3	21,708
	Anacondas^s^	0.3	50,856	2.3	21,708

*In relation to the estimated pooled records of dead animals (small vertebrates plus medium to large vertebrates.

**In relation to the estimated number of dead animals in the small vertebrates and the medium to large vertebrate groups, separately.

^s^Subgroups of vertebrates considered as underestimated (see "Discussion").

## Discussion

The estimated numbers indicate an astonishing immediate impact of the Pantanal’s 2020 wildfires in the vertebrate communities, even considering that the estimates do not reflect the complete figure of mortality, as hidden (e.g., underground), delayed or second order effects certainly caused an unknown number of deaths. In fact, late mortality may be caused not only by body burns but also due to changes in the vegetation and the consequent impacts on resource quality, availability, and productivity at every trophic level, ultimately leading survivors to starvation^31–35^. Mortality also may occur due to increased predation during displacement from affected home ranges^31,36^. The negative consequences may be stronger for small populations or species that require more time to recover^36–38^^)^. Thus, the overall impact of the catastrophic wildfire that hit the Pantanal in 2020 on the vertebrate communities should be considered as substantially higher than our estimates of the direct mortality by the fire.

Our surveys missed several species known to have been killed by wildfires in the Pantanal, which figured in the news media or were reported by collaborators and firefighters. Among these species were large-bodied animals usually living at relatively low densities, such as the jaguar (*Panthera onca*), the puma (*Puma concolor*), the lowland tapir (*Tapirus terrestris*), as well as species such as the red-brocket deer (*Mazama americana*), the giant anteater (*Myrmecophaga tridactyla*), the marsh deer (*Blastocerus dichotomus*), the pampas deer (*Ozotoceros bezoarticus*), the collared peccary (*Pecari tajacu*). Other common carnivores not detected in our surveys were the crab-eating fox (*Cerdocyon thous*), the racoon (*Procyon cancrivorus*), the maned wolf (*Chrysocyon brachyurus*), the tayra (*Eira barbara*), ocelot (*Leopardus pardalis*), the coati (*Nasua nasua*), and the jaguarundi (*Herpailurus yagouaroundi*), among other species known to occur in the Pantanal^39,40^. Large-bodied vertebrates have been among the injured animals frequently found alive by rescuers after the fires in the Pantanal, indicating that they may be less prone to die immediately. Large animals may die days and weeks after the fire due to burns, and this may explain the fact that species such as tapir, marsh deer, and jaguars were not detected in our surveys. In our surveys, medium to large animals were detected at larger distances from the transect line if compared with smaller animals, indicating that our decision on splitting the data set in these two groups was correct. Conceptually, the effective sample trip is defined by the distance from the transect line in which the number of animals detected beyond such distance equals the number of animals missed within this distance, and it is used to estimate densities^41^.

Indirect estimates for the Australian savannas suggest that nearly 15,780 vertebrates per km^2^ were affected by the 2019/2020 bushfires, due to direct mortality and displacement, starvation, habitat loss and impoverishment, among other indirect effects of fire^28^. Similar exercise conducted in the Pantanal for the 2020 wildfire resulted in approximately 1,710 affected vertebrates per km^2^ (at least 65 million native vertebrates) plus 4 billion invertebrates in 38,000 square kilometers^30^. In Bolivia, researchers adopted an expert-based approach to determine mortality rates due to fire and theoretical estimates of mammal population densities, suggesting that 295.7 mammals per km^2^ (5.9 million individuals) were killed by the 2019 wildfires that affected nearly 20,000 km^2^ of the Chiquitano Dry Forest^29^. Although these assessments may be considered valid attempts of estimating the impact of wildfires, they are not based on direct carcass counts, they do not account for the extremely variable landscape composition, vegetation biomass, and flammability among regions hit by these events. Comparisons among these numbers and the results we present in this article may be virtually impossible as methods also varied among these studies. It is important to highlight that our estimates for the Pantanal are certainly underestimates for some taxonomic groups. Among the expected undetected dead animals in the surveyed areas in the Pantanal we may include especially amphibians, snakes, rodents, and armadillos, among other animal groups composed of fossorial animals and wood hollow users. Many small-bodied species may have died in places where they can not be accounted for, or their bodies may have been completely calcined or covered by ash. However, as our estimates are based on direct carcass counts, and they may be considered a better approximation of the reality when compared with theoretically based estimates of densities and/or mortality due to wildfire.

Estimating the number of deaths among wildlife species due to fire is relevant to contribute to the discussion on the potential cumulative impact of recurrent wildfires on ecosystems, as extensive fires compose a plausible scenario under climate change worldwide^1–4,42^. Indeed, the climate change scenarios for the Pantanal region indicate a 30% rainfall decrease in relation to the average precipitation between 2070 and 2100, as well as an increase in temperature and frequency of the extreme climatic events^20^. However, there was already a 40% shortage in rainfall in the region in 2020^19^, as well as an increase of 2 °C in the average temperature since 1980^18^, creating the ideal conditions that contributed to the 2020’s wildfires in the Pantanal wetland. In fact, there is a trend occurring in the Pantanal, as a 376% increase in annual average burned area has been registered for the last two decades, with 43% of the area not being previously burned during this period^6^. Drought variability in the Pantanal region is closely related to teleconnection patterns associated with sea surface temperature anomalies in the Atlantic and Pacific oceans^43^. Despite the extreme droughts in the Pantanal region seem to be linked to a temperature anomaly in the tropical Atlantic ocean^19,43,44^, there is a negative synergy between the extreme climatic event with locally inadequate human factors in causing uncontrolled fire^15,45^. The complicated perspective for the future of the Pantanal wetland also relies on the fact that moisture from the Amazonian rainforest plays a significant role in controlling summer rainfall in central-southern Brazil, including Pantanal^46^. Recent studies show that deforestation in the rainforest is connected to lower transfer of moisture for the Pantanal wetland^47^. Since deforestation and fire are increasing again in the Amazon rainforest^15^, a challenging scenario for the Pantanal also includes other contributing factors such as hydrological changes due to river damming^48–51^, soil erosion and deforestation in surrounding plateaus^52,53^, which can cause wetland area losses, exposing more areas to the risk of fire.

Pantanal is already the Brazilian ecoregion with the highest average fire foci per square kilometer^54^. Thus, the perspective of an increased frequency and extension of fire in the Pantanal and other tropical ecosystems poses a serious threat to the conservation of biodiversity and ecosystem services, as the cumulative effects may be considerable in a long-term perspective. In fact, the 2020 wildfires were not limited to the Pantanal wetland, as exceptionally large burning areas were also registered further south, from the vicinity of Buenos Aires in Argentina, crossing into the Chaco region of Argentina, Paraguay and Bolivia and heavily hitting the Chiquitano dry forests of Bolivia, as well as large portions of the Amazon rainforest and the Cerrado savanna in Brazil. Under this perspective, strategies capable of preventing wildfire disasters are key to avoid ecosystem degradation and economic losses, as well as increased emission of greenhouse gases, considering the climate change scenarios. One relevant instrument for such a strategy is the implementation of proper public policies. In Pantanal context, it is important to enact the Integrated Fire Management (IFM) National Bill n. 11,276/2018, which is still under discussion in August 2021 in the Brazilian Chamber of Deputies, drafted with the participation of state agencies, scientists, and traditional and indigenous communities. Also, state-level legislation is relevant as it is more detailed and focused on regional socio-economic, ecological, and cultural context and nuances. In fact, Mato Grosso do Sul state approved a specific policy to regulate the use of fire^55^, and Mato Grosso—the other Brazilian state where the Pantanal wetlands are located—is currently developing its own policy. Both pieces of legislation should serve as the basis for the proper use of fire in the Pantanal, but awareness and training are highly necessary. The basis for these policies is fuel management, which has proven to be an effective way to prevent wildfires and even reduce the risks posed by the combination of biomass, weather, temperature, and drought^56–59^. In these complex environmental, economic, and political scenarios, which are present not only in Brazil and other South American countries, it is likely that ecosystems and entire faunas would suffer from cumulative impact of wildfires in terms of frequency and extent Our results indicate that it is possible to apply standard methods and obtain data capable of supporting impact estimation, as well as trends on wildlife populations exposed to repeated wildfires. We do need consistent data, and large-scale sampling and long-term monitoring should be a priority in ecosystems affected by fire to support the elaboration of conservation and management measures.

As wildfires pose a worldwide threat to ecosystem resilience and overall sustainability, it's worth reinforcing the need for (i) continuous monitoring for early detection of fire risk and fire events; (ii) the establishment of firefighter brigades in strategic locations with continuous operation; (iii) the improvement of logistic capabilities to allow effective access to distant and marshy areas in the floodplain, (iv) community education programs focused on proper fire use for biomass management purposes, (v) effective enforcement of fire policies; and (vi) implementation of wildlife rescue and rehabilitation centers. In the specific case of the Pantanal wetland, for instance, it would be necessary to effectively implement such strategies for fire management in connection with economy, biodiversity conservation, ecosystem management, and public policy, as it has been proposed by sustainability agendas^48^. The case of the Pantanal reminds us that integrated fire management, as well as the implementation of sustainable land use and restoration to mitigate the inevitable impact of climate change are a crucial part of our survival strategy, given our dependency on ecosystems, their biodiversity, and services.

## Methods

### Study area

The Pantanal is a large floodplain located in the Upper Paraguay River Basin, in the center of South America, comprehending 179,4000 km^2^, shared by Paraguay, Bolivia, and Brazil (each one encompassing 4, 18, and 78% of the floodplain, respectively)^48^. Approximately 65% of the Brazilian portion of the Pantanal is located in the Mato Grosso do Sul state, while the remaining 35% is in the neighboring state of Mato Grosso. The Pantanal biodiversity is composed by over 2,000 plant species^60^, as well as by 269 fish^61,62^, 57 amphibian^63,64^, 131 reptile^65^, over 580 bird^66,67^, and at least 174 mammal species^68^. The floodplain is characterized as a wet savanna, in which the landscape is composed by a mosaic of forests, open woodland savanna, non-floodable grasslands, seasonally flooded grasslands, and aquatic habitats (freshwater ponds, brackish water ponds, oxbow lakes, large lakes, seasonally flooded grasslands, intermittent or seasonally running channels, rivers and swamps). A network of protected areas covers less than 5% of the floodplain, and comprises one National Park, one Ecological Station, three State Parks, and several Private Reserves of Natural Heritage^48^. Most of the Pantanal is considered by UNESCO as a Biosphere Reserve (the third largest in the world), as well as a National Heritage according to the Brazilian Constitution. Over 80% of the original landscapes in the Pantanal are still conserved, and the main economic activity is the extensive cattle ranching^48^. Fire is traditionally used to manage the native grassland and open woodland savannas to improve forage availability for the cattle^24^.

### Survey protocol and data analysis

We conducted post fire line transects to count vertebrates, using the distance sampling technique^41^ to estimate the number of deaths in the 39,030 km^2^ burned region of the Pantanal wetland during 2020 wildfires^17^ (Fig. 1). The transects were placed opportunistically, according to the fire events at different locations between August 1st and November 17th, 2020, as well as timely accessibility and reduction of risks to the field staff. Transects were preferably placed perpendicular to the edge of burned areas, roads, trails, and fence lines. In places without access by such structures, transects started at accessible points, without a pre-established positioning. In this condition, the direction of the transects was defined by the observers, always crossing any type of vegetation found ahead, and avoiding areas that were eventually not burned. The distance between transects was kept at least 200 m, as the subject of the surveys were immobile objects (carcasses). Most of the transects, however, were separated by distances over 500 m. Transects were run by two surveyors, one keeping track of the transect, and the other searching for carcasses and measuring the perpendicular distance to the transect line. To avoid removal of carcasses by scavengers, samplings were conducted within 72 h after burning, but mostly within a 24–48 h period. All field biologists and technicians covering the burned region employed the same standardized sampling protocol. Distance sampling requires the measurement of the perpendicular distance between detected target objects and the transect line, as well as the transect length, to estimate densities based on the curve of detection probability; the premise is that detection probability decreases as the distance from the transect line increases^41^. All dead vertebrates detected along the transect line were identified at least to the Order level, and to lower taxonomic level whenever possible. We used the Distance 7.3 software^68^ to estimate densities and the total number of dead animals, separating them into two groups regarding body size: small vertebrates (less than 2 kg) and medium to large vertebrates (2 kg and over). During the analysis process, we adopted these two groups as strata in the study area, obtaining separate estimates for small and medium to large vertebrates, as well as a pooled estimate for the entire area burned in the Pantanal. The burned area was obtained from the Laboratory for Environmental Satellite Applications (LASA), 2020 (freely available at https://alarmes.lasa.ufrj.br/login). We overlapped the burned area information with surveyed transects by using QGIS software version 3.16.

Based on group and on pooled estimates, we estimated the number of dead vertebrates in subgroups by using the percentage of records of each sub-group in relation to the total number of carcasses recorded in the field, as well as to the number of records by group. The small vertebrate subgroups correspond to amphibians, small lizards, small snakes, small birds, small rodents, and marsupials. Subgroups within the medium to large vertebrates were chelonians, large lizards, anacondas, caimans, medium-large birds, anteaters, armadillos, medium-large rodents, ungulates, and primates, which comprehend the vertebrate species detected in our surveys.

## Supplementary Information

Supplementary Information.

## Data Availability

The data used to conduct the analysis is available at https://doi.org/10.6073/pasta/1688bdf9c001c89972d2cb53d242c4ef (Accessed 2021–08-02).

## References

[1] Chiang F, Mazdiyasni O, AghaKouchak A (2021). Evidence of anthropogenic impacts on global drought frequency, duration, and intensity. Nat. Commun..

[2] Spinoni J, Naumann G, Carrao H, Barbosa P, Vogt J (2014). World drought frequency, duration, and severity for 1951–2010. Int. J. Climatol..

[3] Duane A, Castellnou M, Brotons L (2021). Towards a comprehensive look at global drivers of novel extreme wildfire events. Clim. Change.

[4] Krawchuk MA, Moritz MA, Parisien MA, Van Dorn J, Hayhoe K (2009). Global Pyrogeography: The current and future distribution of wildfire. PLoS ONE.

[5] Williams AP (2019). Observed impacts of anthropogenic climate change on wildfire in California. Earth's Fut..

[6] Garcia LC (2021). Record-breaking wildfires in the world's largest continuous tropical wetland: Integrative Fire Management is urgently needed for both biodiversity and humans. J. Environ. Manag..

[7] Bowman DMJS (2020). Vegetation fires in the Anthropocene. Nat. Rev. Earth Environ..

[8] Criado MG, Myers-Smith IH, Bjorkman AD, Lehmann CER, Stevens N (2020). Woody plant encroachment intensifies under climate change across tundra and savanna biomes. Glob. Ecol. Biogeogr..

[9] Mancini LD, Corona P, Salvati L (2018). Ranking the importance of Wildfires' human drivers through a multi-model regression approach. Environ. Impact Assess. Rev..

[10] Moreira F (2011). Landscape – wildfire interactions in southern Europe: Implications for landscape management. J. Environ. Manag..

[11] Clarke H (2020). The proximal drivers of large fires: A pyrogeographic study. Front. Earth Sci..

[12] Abram NJ (2021). Connections of climate change and variability to large and extreme forest fires in southeast Australia. Commun. Earth Environ..

[13] Daskin JH, Aires F, Staver AC (2019). Determinants of tree cover in tropical floodplains. Proc. R. Soc. B..

[14] Kotze DC (2013). The effects of fire on wetland structure and functioning. Afr. J. Aquat. Sci..

[15] Tedim F (2018). Defining Extreme Wildfire Events: difficulties, challenges, and impacts. Fire.

[16] Libonati, R. *et al*. Sistema ALARMES – Alerta de área queimada Pantanal, situação final de 2020 https://www.researchgate.net/publication/350103205_Nota_Tecnica_012021_LASA-UFRJ_Queimadas_Pantanal_2020?channel=doi&linkId=6051109d92851cd8ce483fb1&showFulltext=true (2021).

[17] Libonati R, DaCamara CC, Peres FL, de Carvalho LAS, Garcia LC (2020). Rescue Brazil’s burning Pantanal wetlands. Nature.

[18] Marengo JA (2021). Extreme drought in the Brazilian Pantanal in 2019–2020: Characterization, causes and impacts. Front. Water.

[19] Marengo JA, Alves LM, Torres RR (2016). Regional climate change scenarios in the Brazilian Pantanal watershed. Clim. Res..

[20] Hardesty J, Myers R, Fulks W (2005). Fire, ecosystems, and people: A preliminary assessment of fire as a global conservation issue. George Wright Forum.

[21] Bliege Bird R, Bird DW, Codding BF, Parker CH, Jones JH (2008). The “fire stick farming” hypothesis: Australian Aboriginal foraging strategies, biodiversity, and anthropogenic fire mosaics. Proc. Natl. Acad. Sci. USA.

[22] Beerling DJ, Osborne CP (2006). The origin of the savanna biome. Glob. Chang. Biol..

[23] Simon MF (2009). Recent assembly of the Cerrado, a neotropical plant diversity hotspot, by in situ evolution of adaptations to fire. Proc. Natl. Acad. Sci. USA.

[24] Pott A, Pott VJ (2004). Features and conservation of the Brazilian Pantanal wetland. Wetl. Ecol. Manag..

[25] Ferraz-Vicentini KR, Salgado-Laboriau ML (1996). Palynological analysis of a palm swamp in Central Brasil. J. South Am. Earth Sci..

[26] Engstrom RT (2010). First-order fire effects on animals: review and recommendations. Fire Ecol..

[27] Whelan RJ, Rodgerson L, Dickman CR, Sutherland EF (2002). Critical life processes of plants and animals: Developing a process-based understanding of population changes in fireprone landscapes.

[28] van Eeden, L. M. *et al*. Impacts of the unprecedented 2019–2020 bushfires on Australian animals. https://www.wwf.org.au/ArticleDocuments/353/WWF_Impacts-of-the-unprecedented-2019-2020-bushfires-on-Australian-animals.pdf.aspx (2020).

[29] Pacheco LF, Quispe-Calle LC, Suárez-Guzmán FA, Ocampo M, Claure-Herrera AJ (2021). Muerte de mamíferos por los incendios de 2019 en la Chiquitania. Ecol. Boliv..

[30] Berlinck CB (2021). The Pantanal is on fire and only a sustainable agenda can save the largest wetland in the world. Braz. J. Biol..

[31] Andersen AN, Woinarski JCZ, Parr CL (2012). Savanna burning for biodiversity: Fire management for faunal conservation in Australian tropical savannas. Austral Ecol..

[32] Komarek R (1963). Fire and the changing wildlife habitat. Proc. Tall Timbers Fire Ecol. Conf..

[33] Layme VMG, Lima AP, Magnusson WE (2004). Effects of fire, food availability and vegetation on the distribution of the rodent *Bolomys lasiurus* in an Amazonian savanna. J. Trop. Ecol..

[34] Roberts SL, van Wagtendonk JW, Miles AK, Kelt DA, Lutz JA (2008). Modeling the effects of fire severity and spatial complexity on small mammals in Yosemite National Park, California. Fire Ecol..

[35] Smith, J. K. *Wildland Fire in Ecosystems: Effects of Fire on Fauna* (Rocky Mountain Research Station, Colorado, 2000).

[36] Woinarski JCZ, Legge S (2013). The impacts of fire on birds in Australia's tropical savannas. Emu.

[37] Pires AS, Fernandez FA, de Freitas D, Feliciano BR (2005). Influence of edge and fire-induced changes on spatial distribution of small mammals in Brazilian Atlantic Forest fragments. Stud. Neotrop. Fauna Environ..

[38] Silveira, L. F., Rodrigues, H. G., Jácomo, A. T. A. & Diniz Filho, J. A. F. Impact of wildfires on the megafauna of Emas National Park, Central Brazil. *Oryx* **33**, 108–114 (1999).

[39] Tomas, W. M. *et a*l. Checklist of mammals from Mato Grosso do Sul, Brazil. *Iheringia, Sér. zool.* **107(Suppl)**, e2017155 (2017).

[40] Tomas WM (2010). Mammals in the Pantanal wetland, Brazil.

[41] Burnham KP, Anderson DR, Laake JL (1980). Estimation of density from line transect sampling of biological populations. Ecol. Monogr..

[42] Jolly WM (2015). Climate-induced variations in global wildfire danger from 1979 to 2013. Nat. Commun..

[43] Thielen, D. Quo vadis Pantanal? Expected precipitation extremes and drought dynamics from changing sea surface temperature. *PLoS ONE* **15(1)**, e0227437 (2020). 10.1371/journal.pone.0227437 PMC6946591 31910441

[44] Ciemer, C. *et al*. An early-warning indicator for Amazon droughts exclusively based on tropical Atlantic Sea surface temperatures. *Environ. Res. Lett.* **15**, 094087 (2020).

[45] Boers N, Marwan N, Barbosa HMJ, Kurths J (2017). A deforestation-induced tipping point for the South American monsoon system. Sci. Rep..

[46] Bergier I (2018). Amazon rainforest modulation of water security in the Pantanal wetland. Sci. Total Environ..

[47] Hofmann G (2021). The Brazilian Cerrado is becoming hotter and drier. Glob. Chang. Biol..

[48] Tomas WM (2019). Sustainability Agenda for the Pantanal Wetland: perspectives on a collaborative interface for science, policy, and decision-making. Trop. Conserv. Sci..

[49] Schulz C (2019). Physical, ecological and human dimensions of environmental change in Brazil's Pantanal wetland: Synthesis and research agenda. Sci. Total Environ..

[50] Harris MB (2005). Safeguarding the Pantanal wetlands: Threats and conservation initiatives. Conserv. Biol..

[51] Ely P, Fantin-Cruz I, Tritico HM, Girard P, Kaplan D (2020). Dam-induced hydrologic alterations in the rivers feeding the Pantanal. Front. Environ. Sci..

[52] Roque, F. O. *et al*. Simulating land use changes, sediment yields, and pesticide use in the Upper Paraguay River Basin: Implications for conservation of the Pantanal wetland. *Agric. Ecosyst. Environ.* **314**, 107405 (2021).

[53] Guerra, A. *et al*. Drivers and projections of vegetation loss in the Pantanal and surrounding ecosystems. *Land Use Policy* **91**, 104388 (2020).

[54] Berlinck, C. N., Lima, L. H. A. & Carvalho Junior, E. A. R. Historical survey of research related to fire management and fauna conservation in the world and in Brazil. *Biota Neotropica* **21(3)**, e20201144 (2021).

[55] Estado de Mato Grosso do Sul. DECRETO Nº 15.654, de 15 de abril de 2021. Institui o Plano Estadual de Manejo Integrado do Fogo, e Dá Outras Providências. (Diário Oficial do Estado, Mato Grosso do Sul nº 10.477, 2021).

[56] Marino E (2014). Forest fuel management for wildfire prevention in Spain: A quantitative SWOT analysis. Int. J. Wildland Fire.

[57] Finney, M. A. & Cohen, J. D. *Expectation and Evaluation of Fuel Management Objectives* (Rocky Mountain Research Station, Colorado, 2003).

[58] Amiro BD, Stocks BJ, Alexander ME, Flannigan MD, Wotton BM (2001). Fire, climate change, carbon and fuel management in the Canadian boreal forest. Int. J. Wildland Fire.

[59] Rocca ME, Brown PM, MacDonald LH, Carrico CM (2014). Climate change impacts on fire regimes and key ecosystem services in Rocky Mountain forests. Forest Ecol. Manag..

[60] Pott VJ, Pott A, Lima LCP, Moreira SN, Oliveira AKM (2011). Aquatic macrophyte diversity of the Pantanal wetland and upper basin. Braz. J. Biol..

[61] Britski, H. A., Silimon, K. Z. S. & Lopes, B. S. *Peixes do Pantanal: Manual de Identificação* (EMPRAPA, Brasília, 2007).

[62] Sousa TP (2017). Cytogenetic and molecular data Support the occurrence of three Gymnotus species (Gymnotiformes: Gymnotidae) used as live bait in Corumbá, Brazil: Implications for conservation and management of professional fishing. Zebrafish.

[63] Piva A, Caramaschi U, Albuquerque NR (2017). A new species of Elachistocleis (Anura: Microhylidae) from the Brazilian Pantanal. Phyllomedusa.

[64] Strüssmann, C., Ribeiro, R. A. K., Ferreira, V. L., & Beda, A. D. F. *Herpetofauna do Pantanal Brasileiro [Herpetofauna of the Brazilian Pantanal]*. (Sociedade Brasileira de Herpetologia, Belo Horizonte, 2007).

[65] Ferreira, V. L. *et al*. Répteis do Mato Grosso do Sul [Reptiles from Mato Grosso do Sul]. Brazil. *Iheringia Sér. Zool.* **107(Suppl)**, e2017153 (2017).

[66] Nunes AP (2011). Quantas espécies de aves ocorrem no Pantanal? [How many bird species do occur in the Pantanal?]. Atualidades Ornitológicas.

[67] Tubelis DP, Tomas WM (2003). Bird species of the Pantanal wetland, Brazil.. Ararajuba.

[68] Thomas L (2010). Distance software: design and analysis of distance sampling surveys for estimating population size. J. Appl. Ecol..

