# Supplementary material for: Distance sampling surveys reveal 17 million vertebrates directly killed by the 2020’s wildfires in the Pantanal, Brazil
Source: Sci Rep. 2021 Dec 16;11:23547. doi: 10.1038/s41598-021-02844-5 (PMC8677733; doi:10.1038/s41598-021-02844-5)
Supplement: Supplementary file 1 — Supplementary Information. [file 41598_2021_2844_MOESM1_ESM.pdf]

Supplementary information

S1. Identified vertebrate species detected along transect lines, directly killed by the 2020's wildfire in the Brazilian portion of Pantanal wetland. Groups refer to small (S) and medium to large (L) bodied vertebrates.

Group	Sub-group	Identified species
S	Amphibians	*Leptodactylus luctator*
		Leptodactylus macrosternum
		Pseudis platensis
		Rhinella diptycha
		Scinax acuminatus
	Small snakes	*Chironius dixoni*
		Chironius laurenti
		Helicops boitata
		Helicops leopardinus
		Hydrops caesurus
		Liophis militaris
		Palusophis bifossatus

	Pseudoeryx plicatilis
	Amerotyphlops sp.
Small lizards	*Copeoglossum nigropunctatum*
	Manciola guaporicola
	Ophiodes sp.
Small birds	*Chionomesa fimbriata*
	Columbina talpacoti
	Crotophaga ani
	Cyanocorax chrysops
	Leptotila rufaxilla
	Leptotila verreauxi
	Picumnus albosquamatus
	Procacicus solitarius
	Ramphastos toco
	Sporophila collaris
	Tinamidae

	Small marsupials	*Cryptonanus chacoensis*
		Philander canus
	Small rodents	*Holochilus chacarius*
		Sylvilagus brasiliensis
L	Large lizards	*Dracaena paraguayensis*
		Iguana iguana
	Anacondas	*Eunectes notaeus*
	Chelonians	*Chelonoidis carbonarius*
	Caiman	*Caiman yacare*
	Medium to large birds	*Aramides cajaneus*
		Aramus guarauna
		Ciconiidae
		Crax fasciolata
		Crypturellus undulatus
		Ortalis canicollis
		Penelope ochrogaster
	Armadillos	*Dasypus novemcinctus*
		Euphractus sexcinctus
	Anteaters	*Tamandua tetradactyla*
	Primates	*Alouatta caraya*
		Plecturocebus cf. *pallescens*

	Sapajus cay
Medium to large rodents	*Coendou prehensilis*
	Dasyprocta azarae
	Hydrochoerus hydrochaeris
Ungulates	*Mazama americana*
	Tayassu pecari
